# Distinct N7-methylguanosine profiles of circular RNAs in drug-resistant acute myeloid leukemia

**DOI:** 10.1038/s41598-023-41974-w

**Published:** 2023-09-07

**Authors:** Jinqiu Fu, Libo Si, Yao Zhou, Dong Li, Ran Wang

**Affiliations:** 1 Department of Pediatrics, Qilu Hospital of Shandong University, Jinan, China; 2 Department of Thoracic Surgery, Qilu Hospital of Shandong University, Jinan, China; 3 Department of Hematology, Qilu Hospital of Shandong University, Jinan, Shandong Province China

**Keywords:** Cancer, Computational biology and bioinformatics, Medical research

## Abstract

Post-transcriptional methylation modifications, such as the N7-methylguanosine (m7G) modification, are increasingly acknowledged for their role in the development and resistance to chemotherapy in acute myeloid leukemia (AML). This study employed MeRIP-seq technology to investigate the m7G sites within circular RNAs (circRNAs) derived from human AML cells and drug-resistant AML cells, in order to identify these sites more comprehensively. In addition, a detailed analysis of the relationship between m7G and drug-resistant AML was conducted. The bioinformatics analysis was utilized to predict the functions of specific methylated transcripts. The findings revealed a significant difference in m7G level between AML cells and drug-resistant AML cells, suggesting a potentially critical role of m7G in circRNAs in drug-resistant AML development. The methylation of M7G could affect the circRNA-miRNA-mRNA co-expression during the development of AML resistance, which could further influence the regulation of resistance-associated target genes in AML. Furthermore, gene ontology analysis indicated that the distinct distribution pattern of circRNAs with m7G methylation in drug-resistant AML cells was correlated with metabolism-related pathways. These results suggested a potential association between drug-resistant AML and m7G methylation of circRNAs. Moreover, the results revealed a novel role of m7G RNA methylation in circRNAs in the progression of AML chemoresistance.

## Introduction

Acute myeloid leukemia (AML) is a hematological tumor with a relatively poor prognosis^1^. The treatment of AML has traditionally relied on strong chemotherapy drugs, killing cancer cells. However, the prognosis of AML treatment fluctuates depending on the molecular biology expressed by the patient^2^. Therefore, understanding the molecular and phenotypic characteristics of AML plays a crucial role in its initial presentation and requires a thorough understanding of its underlying molecular mechanisms. This knowledge is essential for identifying novel prognostic biomarkers and identifying therapeutic targets, thereby preventing disease recurrence and improving long-term outcomes.

Primary or secondary resistance, including both intrinsic and acquired resistance, plays a crucial role in limiting the prognosis of patients with AML^3^. Abnormal activation of drug-resistant signaling pathways caused by genetic alterations can lead to drug resistance of AML^4^. Initially believed to be a byproduct of faulty alternative splicing, circRNAs, also known as non-coding RNAs (ncRNAs), have emerged as a distinct class^5,6^. Due to significant advancements in high-throughput sequencing methods, it has been found that circRNAs play a vital role in various types of cancer, including AML. For instance, circRNAs have emerged as valuable biomarkers for the diagnosis and prognosis of AML. Previous studies have demonstrated that circRNAs can exert their influence on cancer progression by regulating translational, post-transcriptional, and transcriptional gene expressions^7,8^. Notably, a recent study revealed that a subset of circRNAs, previously deemed untranslatable, can undergo translation following RNA methylation^9^. Nevertheless, our understanding of the extent of RNA methylation, including circRNA methylation, in resistant cells of AML is limited. To address this knowledge gap, scholars have explored the involvement of ncRNAs, particularly circRNAs, in drug resistance of AML, facilitating the development of innovative therapeutic approaches to overcome this challenge^5^.

In the present study, a comprehensive analysis was performed on N7-methylguanosine (m7G) modifications of circRNAs in drug-resistant AML cells. The findings indicated distinct differences in the methylation levels and patterns between drug-resistant AML cells and AML cells. Specifically, the methylation levels in drug-resistant AML cells were significantly higher compared to those in AML cells. Moreover, the differentially methylated genes were derived from various chromosomes, highlighting the widespread impact of these modifications. The results strongly indicated a potential association between AML drug-resistance and m7G modifications of circRNAs.

## Materials and methods

### Cell lines and cell culture

Cells were provided by the American Type Culture Collection (ATCC). Cell culture was performed using an RPMI-1640 medium supplemented with thermally inactivated fetal bovine serum (10%) and antibiotics (1% penicillin–streptomycin in v/v). The cultures were maintained at a temperature of 37 °C in an atmosphere consisting of 5% CO_2_ and high humidity (15).

### Cell sensitivity to mitoxantrone

The cell sensitivity to mitoxantrone was assessed using Cell Count Kit-8 (CCK-8) reagent. HL60 and HL60/MX2 cells (1 × 10^4^ cells/mL) were cultured in 96-well plates and treated with 200 μL of mitoxantrone solutions (range 0.01–2.4 μg/mL) for 24 h. After incubation, 10 μL of CCK-8 reagent was added to each well and the plates were further incubated at ambient temperature with 5% CO_2_ for 120 min. The absorbance at 450 nm was then measured to determine the inhibitory ratio and half-maximal inhibitory concentration (IC50) for each cell line based on their respective concentration/response curves.

### RNA preparation

After collecting cells in the log growth stage, total RNA was extracted using TRIzol reagent, and the RNA concentration was measured using NanoDrop ND-100. The purity of the RNA was assessed by calculating OD260/OD280 ratio. A ratio of 1.8–2.1 was indicative of high RNA purity. To further determine gDNA contamination and RNA integrity, denaturing agarose gel electrophoresis was carried out.

### Sequencing and library construction of RNA MeRIP-seq

It is noteworthy that m7G-IP-Seq was conducted by CloudSeq Inc. Briefly, the Ribo-Zero rRNA Removal kits were utilized to eliminate rRNAs from the total RNA, following the vendor instructions. The TruSeq Stranded Total RNA Library Prep kit was used to construct sequencing libraries, involving RNA pretreatment. The BioAnalyzer 2100 instrument was employed for library quality control and quantification. To adhere to the Illumina sequencing instructions, single-stranded DNA molecules were generated through denaturation of 10 pM libraries. These molecules were then captured on an Illumina flowcell, amplified into clusters in situ, and finally sequenced for 150 cycles using the two-end mode (PE mode) on an Illumina NovaSeq 6000 sequencer.

### Sequence mapping and m7G

Following sequencing, a collection of two-end reads was obtained. To ensure data quality, Cutadapt was employed with a Q30 threshold to remove low-quality reads, retaining high-quality ones. In the investigation of circRNAs within cloud sequence organisms, a comprehensive analysis was conducted with a special concentration on the potential role of m7G. Notably, m7G is a modified form of guanosine, which is characterized by a methyl group at the 7th position. It is commonly present in the 5′ cap structure of RNA molecules and plays a crucial role in various aspects of RNA metabolism, stability, and translation initiation. The presence of m7G modification can influence the interactions of circRNAs with RNA-binding proteins and microRNAs, potentially modulating their functions. The STAR software (ver. 2.5.1b) facilitated the alignment of these high-quality reads, encompassing m7G modification, to the reference transcriptome/genome. Concurrently, the identification and detection of circRNAs were carried out using DCC software (ver. 0.4.4). Leveraging the Circ2Traits and circBase databases, the identified circRNAs, including those possibly bearing m7G-modified bases, underwent annotation. Subsequent steps involved data normalization and differential expression analysis of circRNAs using edgeR software (ver. 3.16.5). The gene set derived from differentially expressed circRNAs was subjected to GO and KEGG pathway enrichment analyses, shedding light on potential functional pathways influenced by m7G-modified circRNAs. This comprehensive analysis provides valuable insights into the intricate roles of m7G modification in the context of circRNA regulation.

### RT-qPCR assay

Total RNAs were extracted from the cells using TRIzol reagent (Invitrogen, Carlsbad, CA, USA). The extracted RNAs were then used to generate cDNAs using SuperScriptTM III Reverse Transcriptase (Thermo Fisher Scientific, Waltham, MA, USA). For RT-qPCR analysis, the cDNAs were subjected to qPCR using the qPCR SYBR Green master mix (GenSeq) in the QuantStudio 5 Real-Time PCR System (Thermo Fisher Scientific). In different cases, ACTB was utilized as the internal control. Data analysis was performed using the 2^−ΔΔCT^ method. The primers that were used in RT-qPCR were summarized as follows: METTL1 forward (F): 5ʹ-AGCTACACGACTGGATGTGC-3ʹ and reverse (R): 5ʹ-TGAGGTGCCTAGATGTCCCA-3ʹ, WDR4 F: 5ʹ-AGAGTTTGTGAGCCGTATCTC-3ʹ and R: 5ʹ-GAAGATGTAGACCACAGGAGTG-3ʹ, RNMT F: 5ʹ-TGGCTGCAAATATGACTTCAAC-3ʹ and R: 5ʹ-CCTGCATTCGTTTTAAGAGCAT-3ʹ, RAM F: 5ʹ-GTCGTATCCAGTGCGTGTCGTG-3ʹ and R: 5ʹ-CACTGATTGGATCGATTGTCAC-3ʹ, WBSCR22 F: 5ʹ-AGCTGTTTTATGACGAGACAGA-3ʹ and R: 5ʹ-GGCAGATAAAGAAGCTCCAATG-3ʹ, ACTB F: 5ʹ-GTGGATCAGCAAGCAGGAGT-3ʹ and R: 5ʹ-AAAGCCATGCCAATCTCATC-3ʹ.

### Joint analysis of transcriptomics and methylome and statistical analysis

The methylome analysis incorporated an in-depth approach to understand the epigenetic landscape. High-quality reads obtained from the sequencing data were meticulously mapped to the human genome (human gencode v32) utilizing the STAR software (ver. 2.5.1b). This process not only facilitated the precise alignment of the reads, but also allowed for the exploration of potential methylation patterns across the genome. Detection and identification of circRNAs, crucial players in the epigenetic landscape, were carried out using the DCC software (ver. 0.4.4). The subsequent stages of analysis involved comprehensive annotation of the identified circRNAs. This annotation process drew from the rich resources of the Circ2Traits and circBase databases. It allowed for a more nuanced understanding of the potential roles of the circRNAs and their interactions with other genetic elements. Following annotation, data standardization was conducted, and differential expression of circRNAs screening was performed using the edgeR software (ver. 3.16.5). This analysis aimed to not only quantify the abundance of circRNAs, but also to ascertain the differences in their expression levels in various conditions. To provide visual insights into the differential expressions of methylated genes, a scatter plot was generated based on the average logCPM (counts per million) of the two groups. This plot served as a graphical representation of the methylation-based differences in gene expression.

Statistical analysis was robustly conducted using GraphPad Prism (GraphPad Software Inc., San Diego, CA, USA) and SPSS (IBM, Armonk, NY, USA) software. The two groups were rigorously compared using Student’s t-test, which is a widely recognized method for assessing the significance of differences between two groups. The resulting *P* values were carefully evaluated, and those less than 0.05 were indicative of statistically significant findings.

### Analysis of the circRNA-miRNA-mRNA (CMM) network

The miRNA binding sites and target mRNAs were visualized using miRanda (ver. 3.3a) and Target Scan (ver. 8.0) software. By comparing the m7G fold enrichment between HL60 and HL60/MX2 cells, a correlation map was generated to investigate the relationship between M7G status and expression. Cytoscape software was utilized to construct a CMM network, consisting of 2 downregulated hypo-methylated circRNAs and 5 upregulated hypermethylated circRNAs.

## Results

### Verification of the two cell lines

**Figure 1:**
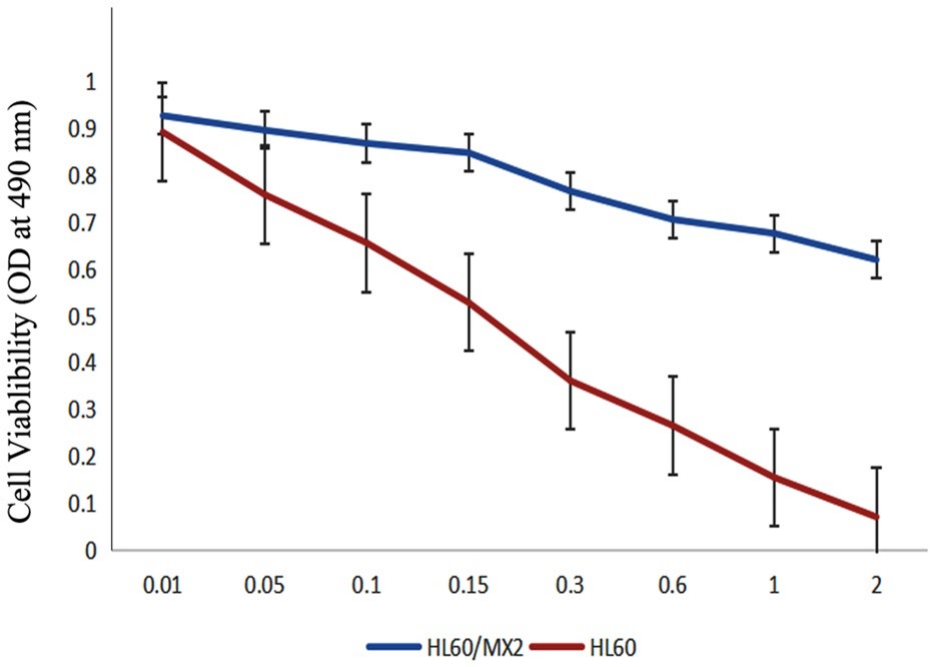
Sensitivity to mitoxantrone in HL60 and HL60/MX2 cells. Mean ± SD (n = 3), *P < 0.05 and **P < 0.01.

HL-60/MX2, a multidrug resistant derivative of HL-60, exhibits resistance to various drugs, including mitoxantrone. In this study, both drug-naive HL60 and drug-resistant HL60/MX2 cells were exposed to different concentrations of mitoxantrone for 24 h. The IC 50 values were then determined. It was revealed that the IC 50 values of HL60 and HL60/MX2 in response to mitoxantrone were 0.15 ± 0.05 and 3.02 ± 0.64 μg/mL, respectively (Fig. 1). These results indicates a noticeable level of resistance in HL60/MX2 towards mitoxantrone.

### Identification of expression profiles of differentially expressed circRNAs in the two cell lines

#### Basic retouching patterns

**Figure 2:**
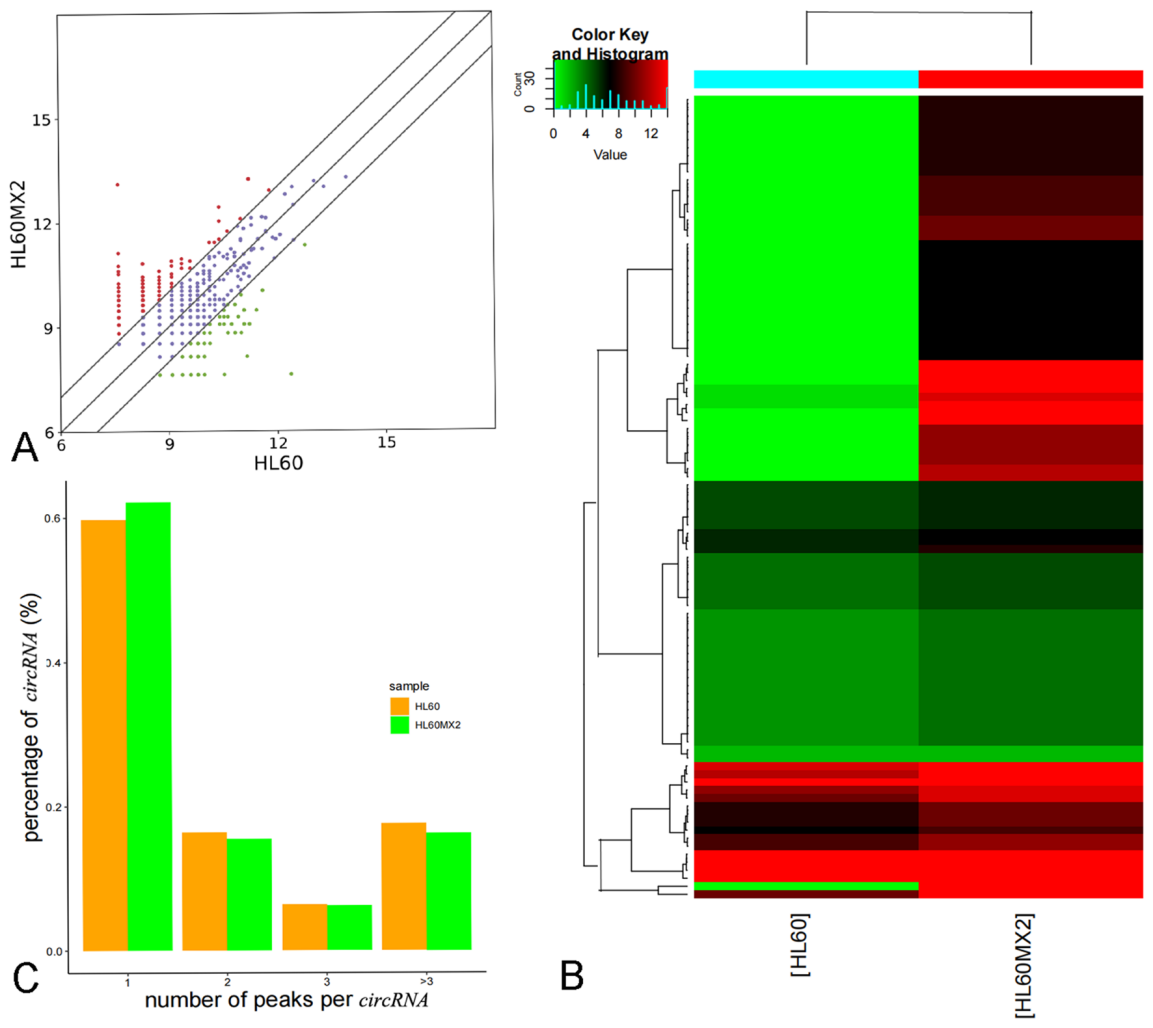
Characterization of expression profiles of circRNAs in HL60 and HL60/MX2 cells. (**A**) Scatter plot reflecting the differences in the expression profiles of circRNAs. The X and Y axes represent normalized average signals that were scaled using a log2 transformation. Purple dots on the graph represent circRNAs, reflecting minimal expression differences (< 2.0-fold change) between the two cell lines. On the other hand, red and green dots indicate circRNAs, exhibiting significant variations (> 2.0-fold change) between the two cell lines. (**B**) A heat map displaying the expression profiles of the top 50 circRNAs that were either upregulated or downregulated in HL60 and HL60/MX2 cells. Each row represents a sample, while each column corresponds to a circRNA. The heat map is color-coded, with red dots indicating high relative expressions and blue dots representing low relative expressions. Notably, three replicates were included in each group. (C) The number of methylation peaks in the two cell lines on each circRNA. As indicated, the majority of circRNAs exhibited a single methylation peak.

The expression profiles of circRNAs in HL60 and HL60/MX2 cell lines were analyzed using the Arraystar Human circRNAs Array. After normalization, 4499 circRNA targets were identified in both cell lines. Filtering based on fold change revealed that 2045 circRNAs exhibited differential expression (*P* < 0.05, fold change > 2) in both cell lines (Fig. 2A). Among these circRNAs, 7933 were upregulated and 1252 were downregulated in HL60/MX2 cells. The top 10 upregulated and downregulated circRNAs are presented in Table 1, while the supplementary Table 1 contains all identified circRNAs. Additionally, a heat map of the top 50 upregulated and downregulated circRNAs (Fig. 2B) highlighted distinctive expression patterns between HL60 and HL60/MX2 cells. These findings suggested that circRNAs could play a role in the drug resistance of AML cells.

**Table 1:** Top 10 upregulated and downregulated circRNAs between HL60 and HL60/MX2 cells as screened by microarray.

Chrom	txStart	txEnd	Gene name	Foldchange
Top 10 up-methylated peaks				
chr7	11021998	11022300	PHF14	3785.4
chr18	12345981	12346460	AFG3L2	2293.7
chr2	36994401	36994428	VIT	1925.4
chr10	75872241	75873100	VCL	1907
chr4	1913541	1914200	WHSC1	1511.1
chr8	120842381	120843080	TAF2	1391.4
chr6	64355561	64355940	PHF3	1271.7
chr6	7304321	7304720	SSR1	1253.3
chr2	100081383	100081447	REV1	1244
chr15	63928159	63928341	HERC1	1161.2
Top ten down-methylated peaks				
chr16	21828782	21828839	RRN3P1	2933.3
chr10	126632161	126632860	ZRANB1	1966.7
chr9	123799601	123800980	C5	1955.9
chr18	44526019	44526180	KATNAL2	1695.2
chr1	76211490	76211599	ACADM	1673.5
chr2	203817501	203817960	WDR12	1467.2
chr2	206930081	206930720	INO80D	1423.7
chr10	74486801	74487200	MCU	1173.9
chr15	63946317	63946340	HERC1	1152.2
chr9	123767861	123768440	C5	1130.5
chr10	126632161	126632860	ZRANB1	1966.7

#### Quantity of peaks on each circRNA

Statistical analysis was conducted on methylation peaks and corresponding circRNAs in order to quantify m7G peaks on each circRNA (Fig. 2C). It was found that the majority of circRNAs in HL60/MX2 cells (62.113%) and HL60 cells (59.697%) containing methylation sites exhibited a single methylation peak. This disparity was found to be statistically significant (*P* < 0.0001). Moreover, HL60/MX2 cells demonstrated a higher abundance of circRNAs with at least two methylation peaks versus HL60 cells (*P* < 0.0001).

#### Sources of circRNAs methylation in the two cell lines

**Figure 3:**
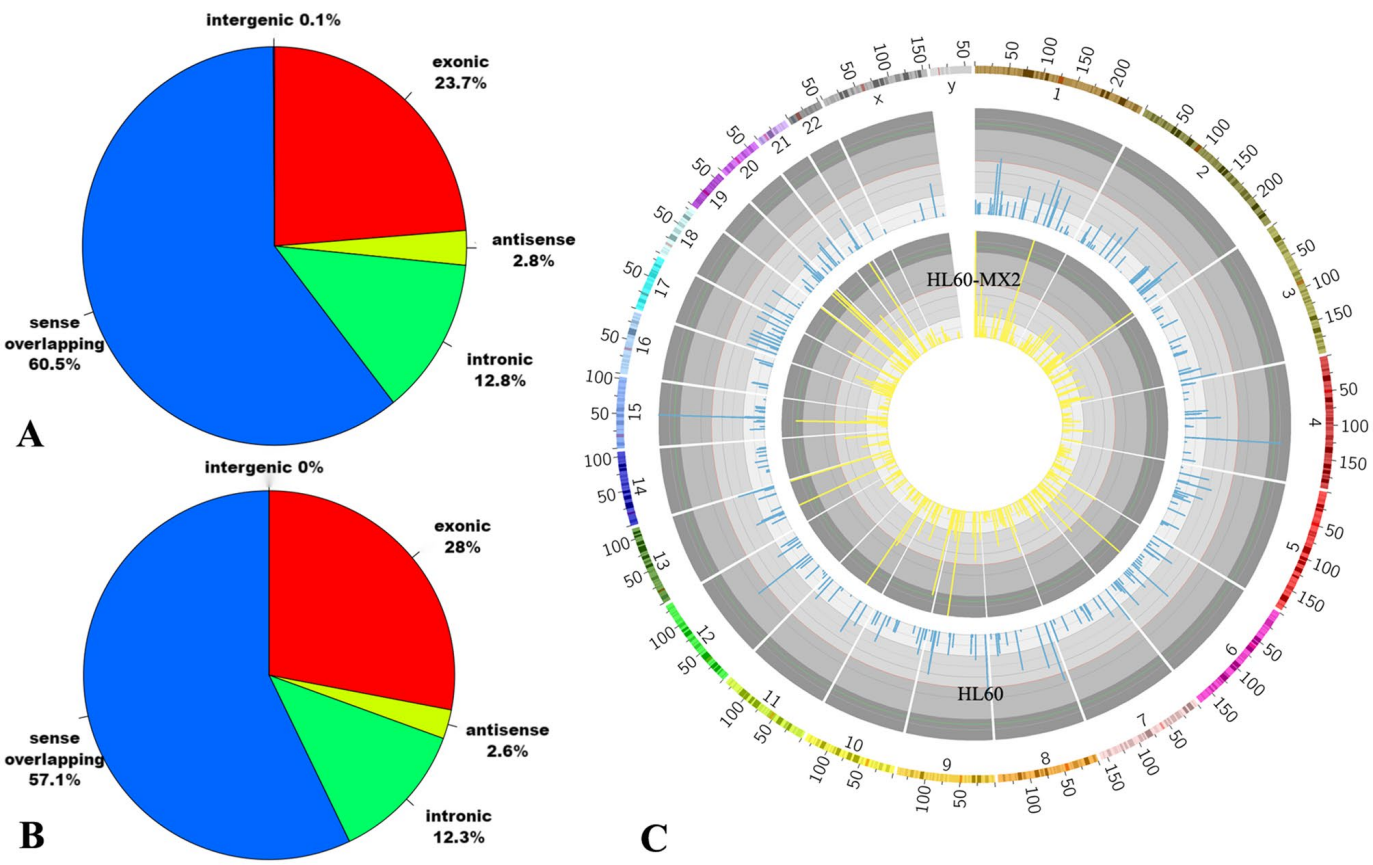
(**A**) and (**B**) Sources of methylated circRNAs in the two cell lines. (**C**) Chromosome-level visualization of m7G in the two cell lines.

The data on methylated circRNAs were analyzed and summarized, and the findings were presented in the form of a pie chart (Fig. 3A,B). The analysis revealed that the majority of circRNAs were derived from sense overlapping regions, followed by exonic regions. In HL60/MX2 cells, there was a higher proportion of methylated circRNAs derived from exons compared with sense overlapping regions, indicating a difference compared with HL60 cells. This demonstrated that the increased quantity of circRNAs derived from exons in HL60/MX2 cells could play a role in influencing cell properties through silencing.

Furthermore, the utilization of Circos enabled visualization of the methylation sites of circRNAs across the chromosomes (Fig. 3C). The results showed differences in methylation sites of circRNAs between the two groups, with the X chromosome and chromosome 14 standing out as particularly notable. It is noteworthy that specific regions on chromosome 14 were associated with various types of leukemia and tumors. Additionally, the analysis revealed that sex chromosomes exhibited lower levels of methylation compared with autosomes in both groups.

#### Motif analysis of methylation sites and cluster analysis of differentially methylated peaks

**Figure 4:**
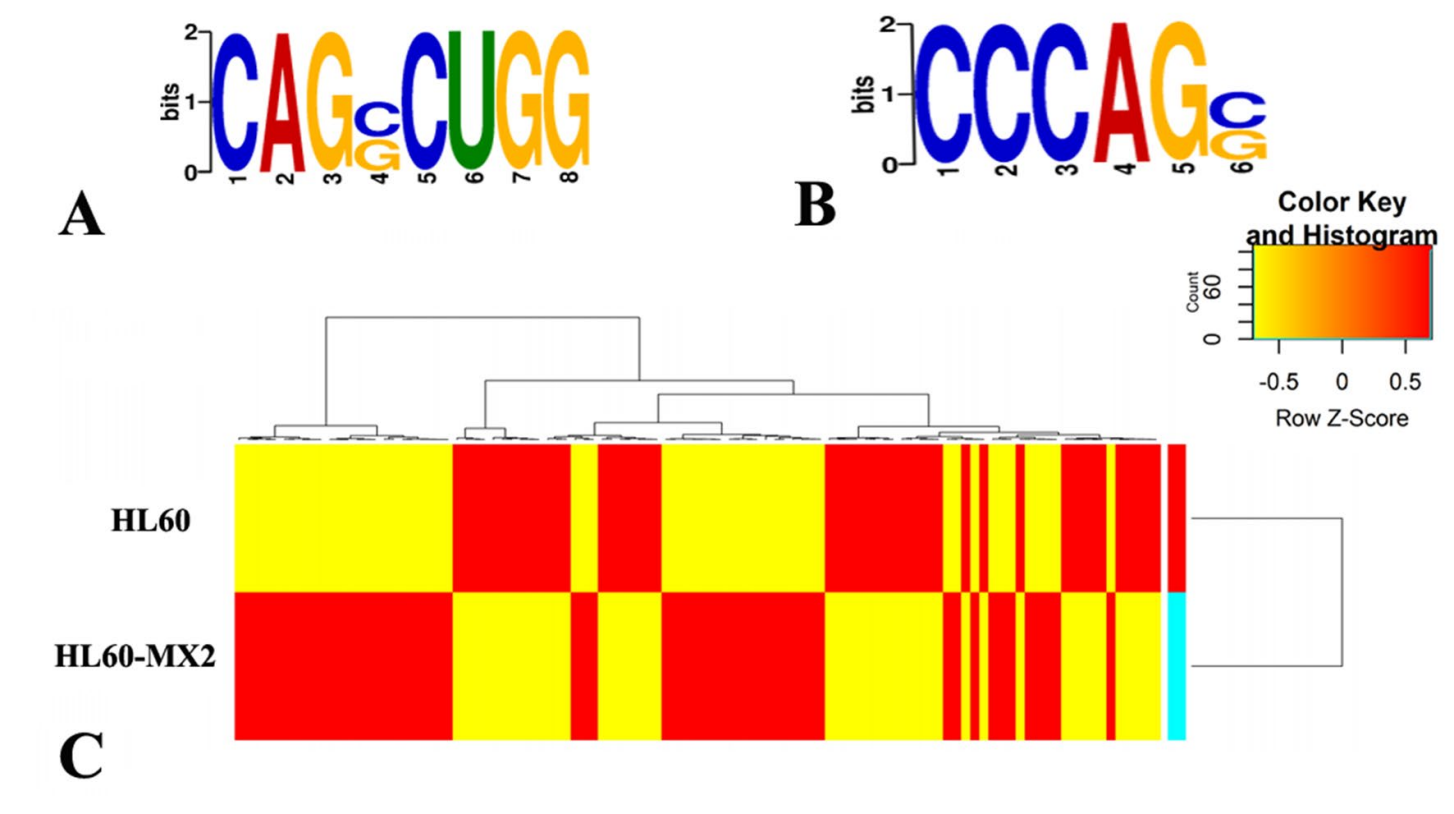
Methylation peaks. Motif sequences of m7G in HL60/MX2 (**A**) and HL60 (**B**) cells (**C**). Cluster analysis of m7G methylation in the two cell lines.

The Dreme was used to scan the sequence of the methylated peak (fifty bp on each side of the apex) in each sample group, and the relevant motif sequences were identified and presented in Fig. 4A,B. Among the motifs measured in the HL60/MX2 cells, the most common and reliable one was CAGSCUGG (S = C/G), indicating conservation (E-value = 1.5e−005). Similarly, in HL60 cells, the most conserved motif was CCCAGS (S = C/G) (E value = 1.4e−004).

Cluster analysis and methylation heatmap analysis were conducted on the total data (Fig. 4C). The results showed that the degree of methylation significantly varied between HL60/MX2 and HL60 cells, indicating significant differences between the two groups, while cells within each group showed relatively consistent methylation patterns. These differences could be attributed to the drug resistance of AML. Specifically, the methylation frequency in HL60/MX2 cells was significantly higher than that in HL60 cells. Furthermore, a total of 102 methylation sites were found to be upregulated in HL60/MX2 cells, while 99 methylation sites were upregulated in HL60 cells.

#### The role of methylation in transcriptional expression

**Figure 5:**
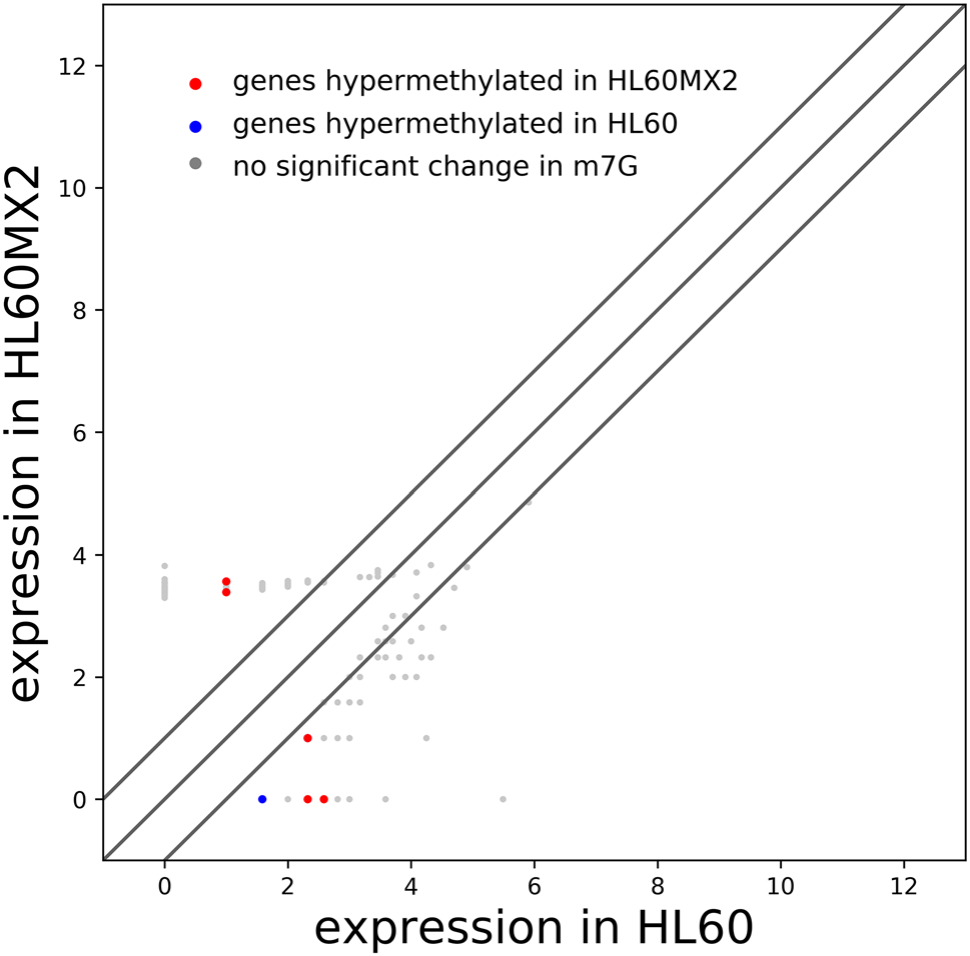
Joint analysis of transcriptome and methylation. Scatter plot reflecting the correlation of gene expression and methylation levels. X- and Y-axis denote the gene expressions in the two cell lines.

To clarify the effects of methylation on transcriptional expression, combined analysis of methylation and transcriptome was conducted (Fig. 5). Table 2 presents the data on differentially methylated RNA sites in HL60/MX2 cells compared with HL60 cells. Notably, seven differentially m7G methylated RNAs were identified that exhibited differential expression of circRNAs (Table 3). Among them, five circRNAs (chr4:187627717–187630999−, chr13:49771013–49772710+, chr6:31237743–31322442−, chr2:45773871–45780869−, and chr18:60206914–60217693+) were upregulated, along with upregulation of mRNA expression levels in FAT1 and SRBD1. Conversely, circRNAs chr10:32740520–32762951+ and chr1:91400965–91406866− showed downregulation, accompanied by downregulation of mRNA expression levels of CCDC7 and ZNF644. Furthermore, downregulated mRNA expression levels of FNDC3A, HLA-C, and ZCCHC2 were found.

**Table 2:** Differences in transcription levels of methylated genes in HL60/MX2 versus HL60 cells.

Differentially methylated RNA	Comparison information		Annotations				
circRNA	Foldchange	Regulation	circBaseID	Gene name	Catalog	logFC	Regulation
chr4:187627717–187630999−	443	Up	hsa_circ_0001461	FAT1	Exonic	2.536408635	Up
chr13:49771013–49772710+	3.836862636	Up	hsa_circ_0005263	FNDC3A	Exonic	− 1.231180961	Down
chr6:31237743–31322442−	148.3	Up		HLA-C	Sense overlapping	− 1.750427248	Down
chr2:45773871–45780869-	719.2	Up	hsa_circ_0005542	SRBD1	Exonic	1.177625667	Up
chr18:60206914–60217693+	682.4	Up	hsa_circ_0000854	ZCCHC2	Exonic	− 1.98394301	Down
chr10:32740520–32762951+	196.5	Down	hsa_circ_0093546	CCDC7	Exonic	− 1.231180961	Down
chr1:91400965–91406866−	99.11764706	Down	hsa_circ_0114424	ZNF644	Intronic	− 1.125843466	Down

**Table 3:** Differentially expressed circRNAs and their association with mRNAs methylated at m7G site.

CircRNA	Regulation	Gene name
Upregulated circRNAs in HL60/MX2 versus HL60 cells		
chr4:187627717–187630999−	Up	FAT1
chr13:49771013–49772710+	Down	FNDC3A
chr6:31237743–31322442−	Down	HLA-C
chr2:45773871–45780869−	Up	SRBD1
chr18:60206914–60217693+	Down	ZCCHC2
Downregulated circRNAs in HL60/MX2 versus HL60 cells		
chr10:32740520–32762951+	Down	CCDC7
chr1:91400965–91406866−	Down	ZNF644

#### GO and KEGG pathway enrichment analyses

**Figure 6:**
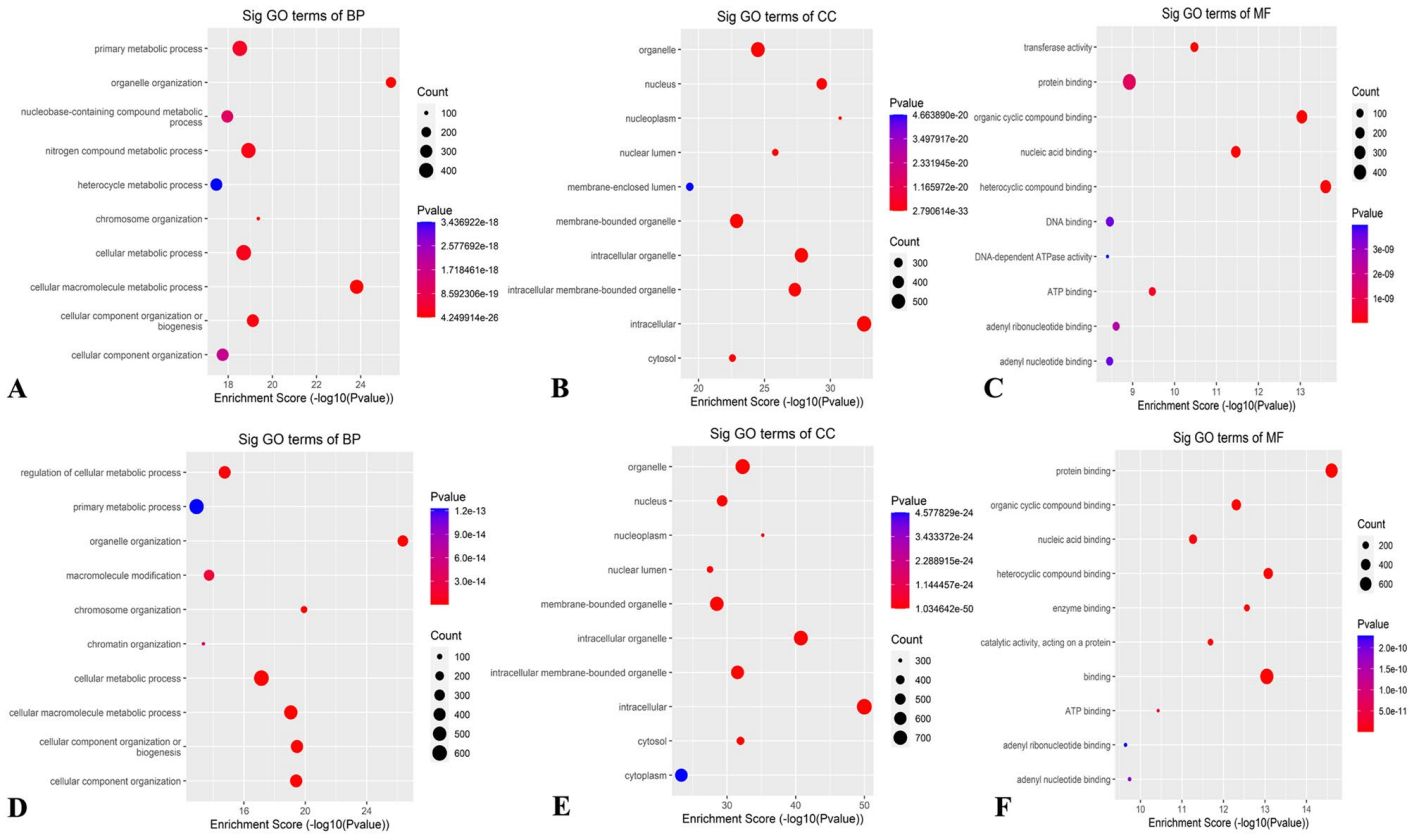
Gene ontology analysis of HL60 and HL60/MX2 cells. (**A**–**C**) BP, CC, and MF in HL60/MX2 cells. (**D**–**F**) BP, CC, and MF in HL60 cells. Top 10 terms were listed in each figure.

Using the data obtained from sequencing, GO analysis was performed to investigate the molecular functions of differentially methylated circRNAs and biological processes in two different cell types. In terms of biological processes (BP), the genes corresponding to up-methylated m7G sites in HL60/MX2 cells were primarily involved in nitrogen compound metabolic processes, cellular metabolic processes, and primary metabolic processes. On the other hand, the genes corresponding to down-methylated m7G sites were primarily involved in cellular component organization or biogenesis, cellular macromolecule metabolic processes, and metabolic processes. When considering cellular components (CC), the upregulated methylation sites in HL60/MX2 were enriched in membrane-bounded organelles, intracellular membrane-bounded organelles, and intracellular organelles, similar to the pattern observed in HL60 cells. In terms of molecular functions (MF), it was found that genes with hypermethylated m7G were primarily associated with heterocyclic compound binding, organic cyclic compound binding, and protein binding. Conversely, genes with downmethylated m7G were primarily associated with heterocyclic compound binding, organic cyclic compound binding, and protein binding. The top 10 prominent categories are depicted in Fig. 6A–F.

**Figure 7:**
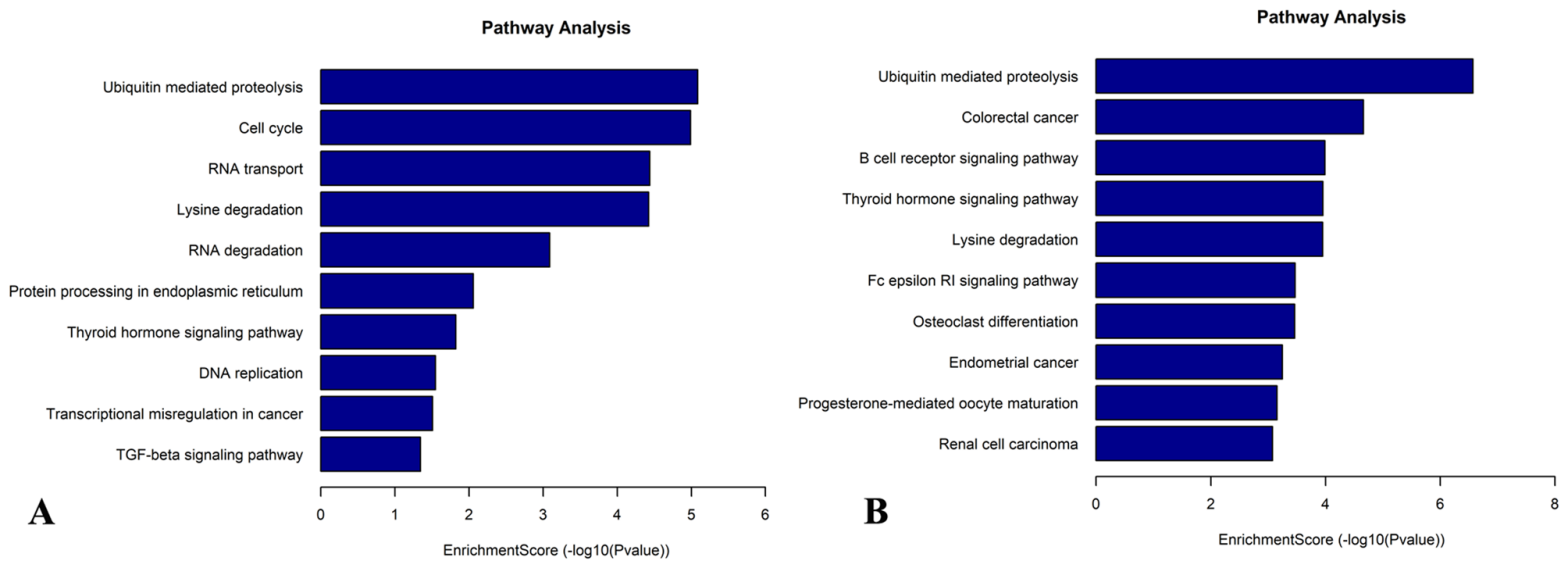
KEGG pathway analysis of differentially methylated genes in the two cell lines. Pathway analysis of differentially methylated genes in HL60/MX2 (**A**) and HL60 (**B**) cells. Top 10 terms were listed in each figure.

The KEGG pathway analysis was employed to identify pathways potentially associated with genes exhibiting differential methylation (Fig. 7A,B). The analysis revealed that genes showing increased methylation in HL60/MX2 cells were involved in proteolysis mediated by ubiquitin, cell cycle regulation, and RNA transport. On the other hand, genes with decreased methylation at m7G were found to be associated with proteolysis mediated by ubiquitin, B cell receptor signaling pathway, and thyroid hormone signaling pathway.

#### Internal m7G sites are installed by methyl transferase

**Figure 8:**
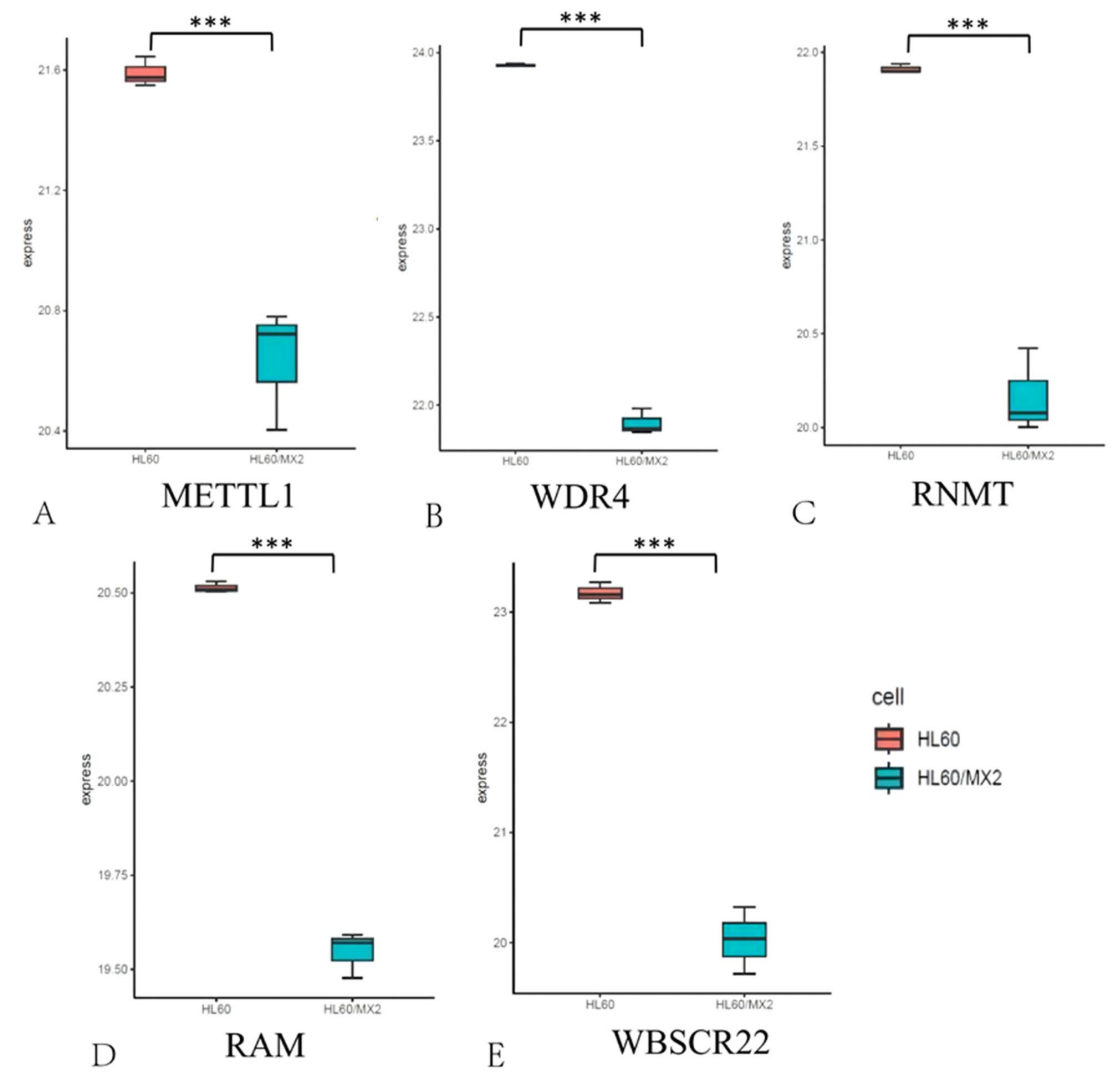
The mRNA levels of methyl transferases METTL1 (**A**), WDR4 (**B**), RNMT (**C**), RAM (**D**), and WBSCR22 (**E**) in HL60/MX2 and HL60 cell lines were determined by RT-qPCR analysis. Data were presented as mean ± SD (n = 3). ***P < 0.001.

In order to further assess the effects of methyl transferase on resistant AML cells, RT-qPCR assay was employed for detecting the mRNA levels of methyl transferase in HL60/MX2 and HL60 cell lines. Compared with HL60 cells, the mRNA levels of METTL1, WDR4, RNMT, RAM, and WBSCR22 were significantly reduced in HL60/MX2 cells (Fig. 8).

#### Co-expression network of CMM in AML

**Figure 9:**
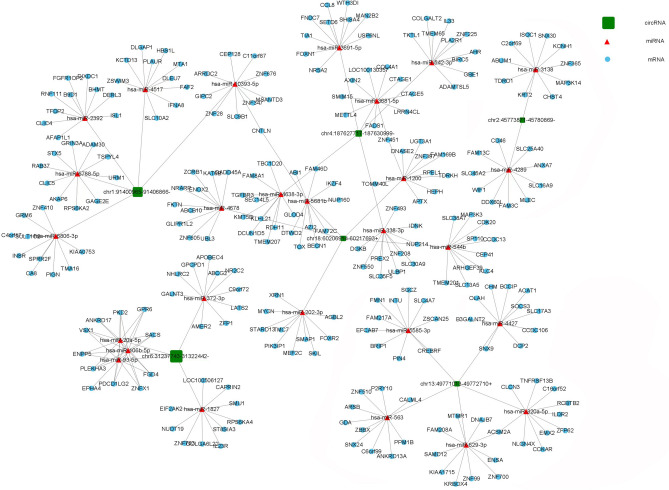
The circRNA-miRNA-mRNA networks in AML. CeRNA analysis concentrated on circRNAs with differential expression, along with m7G methylated RNAs. In the networks, circRNAs were represented by green rectangle junctions, microRNAs by red triangle junctions, and mRNA by blue circular junctions.

To construct the CMM network, seven circRNAs that exhibited differential expression of m7G methylated RNAs were identified. Among these circRNAs, five were found to be hyper-upregulated and two were hypo-downregulated. The specific circRNAs are summarized in Table 2. This network provides valuable insights for future research on the underlying mechanisms of circRNAs in AML tumorigenesis and resistance. The ceRNA network analysis revealed that the seven hyper-upregulated circRNAs were associated with 22 miRNAs, while regulating several mRNAs. The top 10 regulated mRNAs are displayed in Fig. 9. Additionally, the hypo-downregulated circRNA chr1:91400965–91406866− was found to interact with five miRNAs and regulate multiple mRNAs, which were also identified as the top 10 in Fig. 9. On the other hand, the hypo-downregulated circRNA chr10:32740520–32762951+ did not exhibit any association with miRNAs. However, through the GEPIA database, it was found that the regulated mRNAs were specifically correlated with AML. This suggested that m7G could play a role in the regulation of AML-resistant genes through the CMM co-expression network.

## Discussion

Drug-resistant AML cells display distinct cytogenetic and molecular characteristics, mainly resulting from the deletion or activation of multiple pathways associated with drug resistance^3^. A key contributor to drug resistance in cancer is m7G, which is recognized as a specific type of methylation pathway^10^. Notably, the quantity of m7G sites in drug-resistant AML cells is significantly greater than that in AML cells, and its down-methylated m7G modification is specifically enriched in ABC transporter-related mRNAs, which are known to contribute to multidrug resistance^11^. Despite m7G’s crucial role in cells, the presence and function of its circRNAs in AML cells with drug resistance have not been explored, leaving a gap in understanding of the mechanism underlying AML drug resistance.

In the present study, MeRIP-seq was employed to sequence the m7G methylation of circRNAs in HL60/MX2 and HL60 cells, comparing the results between the two groups. The findings revealed the identification of various methylation peaks, with a significant disparity in both the quantity and distribution of these peaks. Notably, it was found that the genes and methylation frequency in HL60/MX2 cells were substantially higher than those in HL60 cells, indicating a clear association between m7G and resistant AML. Furthermore, the analysis at the whole chromosome level demonstrated significant discrepancies in circRNAs from different chromosomes in both cell types, particularly the X chromosome. This suggested extensive alterations in m7G methylation in HL60/MX2 cells, potentially involving multiple pathways that could influence drug resistance of AML cells. Moreover, the results of motif analysis revealed that the sequences close to the methylation sites were consistent and relatively conserved in both groups, demonstrating that the types of methylation were relatively constant, whereas their quantities varied.

In the present study, it was revealed that PHF14, among the upregulated circRNAs, was associated with the development and progression of various malignancies. Knockdown of PHF14 in colorectal cancer cells may potentially inhibit the carcinogenic process in vivo^12^. Moreover, ZRANB1, a downregulated circRNA, has been found to be closely associated with cancer. Suppression of expression level of ZRANB1 has been found to inhibit the growth and metastasis of hepatocellular carcinoma both in vitro and in vivo. Higher expression level of ZRANB1 was associated with poorer survival^13^. Thus, we hypothesized that these altered circRNAs, influenced by changes in m7G methylation levels, might play a role in chemoresistance in AML.

Previous studies have demonstrated that chromosomal translocation in cancer could lead to the production of abnormal fused circRNAs, which were strongly associated with drug resistance^14,15^. Analysis of bone marrow samples from patients diagnosed with refractory and recurrent AML revealed the upregulated expression level of circPAN3. It was found that downregulation of circPAN3 could decrease the expression level of X-linked inhibitor of apoptosis protein (XIAP). However, this effect was counteracted by specific inhibitors of miR-183-5p or miR-153-3p, suggesting that circPAN3 could be a crucial mediator of chemoresistance in AML cells^16^. In addition, silencing of circMYBL2 was found to enhance differentiation and suppress proliferation of FLT3-ITD AML cells both in vitro and in vivo. CircMYBL2 was noted to increase the translational efficiency of FLT3 kinase by facilitating the binding of polypyrimidine tract-binding protein 1 to FLT3 messenger RNA^17^. Furthermore, the present study demonstrated that only a small number of methylated circRNAs in AML drug-resistant cell lines were derived from exons. Therefore, it can be concluded that methylation-induced decrease of exon-derived circRNAs leads to suppression of important proteins, ultimately contributing to the development of drug resistance in AML.

Additionally, a correlation was identified between the degree of methylation and gene expression in drug-resistant AML cell lines. In the present study, significant genes were identified that exhibited differential expression in both m7G and the RNA itself. One such gene, FATI, was found to be overexpressed in oxaliplatin-resistant breast cancer tissues and cells. Knockdown of circRNA FATI led to the enhanced apoptosis, decreased migration, and reduced invasion in oxaliplatin-resistant breast cancer cells. This effect was mediated through activation of the Notch and Wnt signaling pathways^18^. Another gene, ZCCHC2, exhibited differential expression in a contradictory manner (up-down regulation). Previous studies have linked ZCCHC2 to immunity and resistance in lymphoma, acute lymphoblastic leukemia, AML, acute promyelocytic leukemia (APL), and chronic lymphocytic leukemia (CLL)^19,20^. ZCCHC2 has exhibited to inhibit retinoblastoma tumorigenesis by suppressing HectH9-mediated K63-linked polyubiquitination and activating c-Myc. Knockout of ZCCHC2, on the other hand, promoted the proliferation of retinoblastoma cells^21^. It was reported that the upregulated gene CCDC7 was intimately associated with the development of cervical cancer. Its expression was found to promote proliferation of cervical cancer cells through the JAK-STAT3 pathway^22^. In the present study, a CMM network was established by integrating expression profiles of matched mRNAs, miRNAs, and circRNAs, demonstrating a positive correlation between mRNA and circRNAs expressions. Additionally, M7G was identified as a mediator of circRNA expression through post-transcriptional modification. By influencing the CMM co-expression networks, it was noted that m7G could play a role in AML resistance, providing a potential mechanism for circRNA activity in AML resistance. Therefore, these circRNAs with differential methylation and expression patterns warrant further investigation to elucidate their molecular function in the emergence and development of drug resistance in AML.

Notably, the results of the present study suggested a correlation among m7G methylations, circRNAs, genes, and their expression with chemoresistance in AML. The expression profiles of circRNAs in two cell lines (HL60 and HL60/MX2) were investigated, and 2045 circRNAs were found to be differentially expressed in both cell lines. Further analysis revealed that the majority of circRNAs in both cell lines had one methylation peak, while the quantity of circRNAs with at least two methylation peaks was higher in HL60/MX2 cells. The source data showed that most of methylated circRNAs were derived from sense overlapping and exonic regions, with a higher quantity of circRNAs derived from exons in HL60/MX2 cells. Motif analysis identified conserved motifs in the methylated peaks in both cell lines. Cluster analysis and methylation heatmap showed differences in methylation degree between HL60/MX2 and HL60 cells, with a higher methylation frequency in HL60/MX2 cells. Methylation was found to affect the transcriptional expression levels of circRNAs, with seven differentially methylated circRNAs showing differential expression. The GO and KEGG pathway enrichment analyses revealed the molecular functions, biological processes, and pathways associated with the differentially methylated circRNAs. Furthermore, mRNA levels of methyl transferase were reduced in HL60/MX2 cells compared with those in HL60 cells. Finally, a co-expression network of circRNAs, miRNAs, and mRNAs involved in AML resistance was constructed, providing insights into the underlying mechanisms of circRNAs in AML chemoresistance. The observed correlation points towards the significance of m7G methylations and circRNAs in influencing gene expression patterns associated with chemoresistance in AML. The altered expression and methylation patterns of circRNAs could contribute to the reduced effectiveness of chemotherapy in AML cells. Overall, the results suggested a complex interplay between m7G methylations, circRNAs, gene expression, and chemoresistance in AML. The differential expression and methylation patterns of circRNAs could potentially be linked to altered gene expressions and cellular responses, contributing to chemoresistance in AML cells. Further research is essential to fully understand the mechanisms underlying these correlations and their implications for AML treatment.

After conducting further bioinformatics analysis and examining the results of GO and KEGG pathway enrichment analyses, differentially methylated genes were identified in the two groups. In particular, it was found that methylated genes in drug-resistant AML cell lines were associated with various cell functions, such as endocytosis, protein metabolism, and energy. This suggests that methylation has a widespread impact on cell function and underscores the need for comprehensive investigation into its underlying mechanisms^23,24^. In the present study, it was discovered that proteolysis mediated by ubiquitin could play a critical role in the resistant process. The ubiquitin–proteasome system (UPS) contributes to regulate the abundance and activity of a wide range of proteins. It also plays a key role in tightly controlling cell cycles, gene expression, cell survival, cell proliferation, and apoptosis^25^. Therefore, bortezomib, a proteasome inhibitor, was approved by the FDA in 2003 for the treatment of multiple myeloma^26^. As ubiquitination has been implicated in oncogenesis, researchers have attempted to explore its role in cancer chemoresistance by investigating the underlying mechanisms. Previous studies have demonstrated that the ubiquitination process is involved in chemoresistance^27^. Blocking the UPS can have several effects, which are summarized as follows: (1) reducing the levels of anti-apoptotic proteins (e.g., tumor necrosis factor-α (TNF-α) and nuclear factor-κB (NF-κB)), (2) increasing the expression level of the pro-apoptotic protein Noxa, (3) activating the caspases-mediated apoptotic pathway, (4) degrading pro-survival proteins and inducing myeloid leukemia cell differentiation protein, and (5) inhibiting drug efflux transporters. In the present study, downregulation of m7G methylation expression was found in ubiquitylation-associated genes (BIRC6, HERC1, and WWP2), demonstrating that m7G methylation in ubiquitylation plays a crucial role in AML resistance.

According to previous research, m7G has been implicated in various cell functions, such as stress response, stem cell function, and cell differentiation^28,29^. Although studies on m7G are still in their preliminary stages, they have provided sufficient evidence regarding its critical role in tumor development. The role of m7G methyltransferase is to introduce m7G modifications in specific positions of target RNA molecules, thereby impacting the production, structure, and maturation of RNA molecules, including mRNAs, miRNAs, and rRNAs. Ultimately, this process regulates the translation process^28^. In AML patients, there is a significant increase in the expression levels of the m7G regulators (METTL1 and WDR4), both at mRNA and protein levels^30^. Besides, stable knockdown of METTL1 could effectively inhibit the growth of leukemia stem cells^30^. Further observations revealed that METTL1-knockout mice had a lower tumor burden and a longer survival than wild-type controls. The mechanism underlying these effects involves the METTL1/WDR4 complex, which enhances the abundance of tRNAs through m7G modification, particularly affecting Arg-TCT-4-1. This modification influences the translation of AGA codon-rich mRNAs by reducing ribosomal pausing efficacy. Thus, the METTL1/WDR4-dominated M7G-modified tRNA drives oncogenic transformation via remodeling mRNA translational activity. This ultimately leads to an amplification in the expression levels of cell-cycle progression genes, making it a potential therapeutic target for AML^30^. Consequently, further research is necessary to fully comprehend the role of m7G in AML.

## Conclusions

In conclusion, the present study clarified the differences in distribution and quantity of m7G between drug-resistant AML cell lines and AML cell lines. The bioinformatics analysis was employed to reveal the distribution and potential function of m7G in drug-resistant AML. Furthermore, the findings demonstrated that m7G methylation could influence the co-expression of CMM in the drug-resistant process of AML, as well as regulating target genes associated with resistance in AML. However, understanding of m7G’s function in cells is still in its early stages. Hence, further research is required to fully explore the role and mechanism of m7G and to identify new therapeutic targets for drug-resistant AML.

### Supplementary Information

Supplementary Table S1.

## Data Availability

The datasets generated and/or analysed during the current study are available in the Gene Expression Omnibus (GEO) repository, https://www.ncbi.nlm.nih.gov/geo/query/acc.cgi?acc=GSE201096.
