# Supplementary material for: Distinct N7-methylguanosine profiles of circular RNAs in drug-resistant acute myeloid leukemia
Source: Sci Rep. 2023 Sep 7;13:14704. doi: 10.1038/s41598-023-41974-w (PMC10485064; doi:10.1038/s41598-023-41974-w)
Supplement: Supplementary file 1 — Supplementary Table S1. [file 41598_2023_41974_MOESM1_ESM.docx]

**S-table 1:** Upregulated and downregulated circRNAs between HL60 and HL60/MX2 cells as screened by microarray.

| Up-methylated peaks | | | | | Down-methylated peaks | | | | |
| --- | --- | --- | --- | --- | --- | --- | --- | --- | --- |
| chrom | txStart | txEnd | Gene name | Foldchange | chrom | txStart | txEnd | Gene name | Foldchange |
| chr4 | 187630101 | 187630300 | FAT1 | 443 | chr12 | 27523141 | 27523163 | ARNTL2 | 1000.2 |
| chr17 | 5223601 | 5223980 | RABEP1 | 360.1 | chr15 | 49543541 | 49543820 | GALK2 | 239.9 |
| chr12 | 124904502 | 124904601 | NCOR2 | 4.801895173 | chr5 | 149400841 | 149401240 | HMGXB3 | 305.1 |
| chr17 | 3836061 | 3836440 | ATP2A3 | 479.8 | chr1 | 76211490 | 76211599 | ACADM | 1673.5 |
| chr10 | 94381141 | 94381230 | KIF11 | 1069.1 | chr2 | 206943881 | 206944360 | INO80D | 1076.2 |
| chr17 | 28038161 | 28038960 | SSH2 | 958.6 | chr1 | 202455901 | 202456300 | PPP1R12B | 544 |
| chr13 | 49772601 | 49772710 | FNDC3A | 3.836862636 | chr11 | 130109706 | 130109791 | ZBTB44 | 370.3 |
| chr15 | 63928159 | 63928341 | HERC1 | 1161.2 | chr5 | 137894241 | 137894440 | HSPA9 | 2.777561788 |
| chr20 | 33075721 | 33076140 | ITCH | 894.2 | chr15 | 63946317 | 63946340 | HERC1 | 1152.2 |
| chr1 | 45167601 | 45168200 | G002087 | 369.3 | chr13 | 50115021 | 50115142 | RCBTB1 | 174.8 |
| chrM | 13846 | 14067 | MTND5 | 2.632052034 | chr12 | 102565981 | 102566380 | PARPBP | 348.5 |
| chr15 | 75705103 | 75705200 | SIN3A | 341.7 | chr19 | 33130261 | 33130394 | ANKRD27 | 435.4 |
| chr8 | 142174441 | 142174900 | DENND3 | 360.1 | chr16 | 69815361 | 69815820 | WWP2 | 696.1 |
| chr3 | 57824321 | 57824820 | SLMAP | 1105.9 | chr6 | 28884221 | 28884580 | TRIM27 | 478.9 |
| chr21 | 30693061 | 30693460 | BACH1 | 765.2 | chr9 | 123767861 | 123768440 | C5 | 1130.5 |
| chr9 | 37355941 | 37356280 | ZCCHC7 | 387.7 | chr10 | 126632161 | 126632860 | ZRANB1 | 1966.7 |
| chr2 | 136519387 | 136519481 | UBXN4 | 4.269276868 | chr12 | 22814081 | 22814139 | ETNK1 | 630.9 |
| chr2 | 61411561 | 61411820 | AHSA2 | 664 | chr8 | 128898021 | 128898220 | PVT1 | 229.1 |
| chr4 | 129839381 | 129839780 | SCLT1 | 498.2 | chr3 | 195103201 | 195103540 | ACAP2 | 2.796371514 |
| chr6 | 31320661 | 31320960 | HLA-C | 148.3 | chr1 | 202470341 | 202470600 | PPP1R12B | 478.9 |
| chr1 | 50988241 | 50988344 | FAF1 | 185.2 | chr3 | 16328121 | 16328500 | OXNAD1 | 565.7 |
| chr10 | 75872241 | 75873100 | VCL | 1907 | chr1 | 202448301 | 202448680 | PPP1R12B | 522.3 |
| chr12 | 102553081 | 102553460 | PARPBP | 9.348214286 | chr11 | 36658621 | 36659640 | C11orf74 | 2.713652045 |
| chrX | 2136021 | 2136420 | DHRSX | 664 | chr11 | 120920101 | 120920660 | TBCEL | 1097.9 |
| chr11 | 36677541 | 36677920 | C11orf74 | 516.6 | chr3 | 57569624 | 57569734 | ARF4 | 1108.8 |
| chrX | 134680304 | 134680394 | DDX26B | 19.51576577 | chr9 | 3382581 | 3382960 | RFX3 | 261.7 |
| chr18 | 12345981 | 12346460 | AFG3L2 | 2293.7 | chr1 | 180961741 | 180962140 | STX6 | 413.7 |
| chr11 | 120917381 | 120917900 | TBCEL | 3.377002288 | chr7 | 24684661 | 24684980 | MPP6 | 348.5 |
| chr2 | 36994401 | 36994428 | VIT | 1925.4 | chr1 | 213035289 | 213035700 | FLVCR1 | 413.7 |
| chr1 | 40320241 | 40320720 | TRIT1 | 5.353811149 | chr18 | 12815381 | 12815820 | PTPN2 | 305.1 |
| chr2 | 232325961 | 232326458 | NCL | 49.610397 | chr16 | 67913541 | 67913677 | EDC4 | 576.6 |
| chr4 | 68661321 | 68661800 | UBA6-AS1 | 838.9 | chr19 | 53591181 | 53591580 | ZNF160 | 370.3 |
| chr5 | 142435592 | 142435651 | ARHGAP26 | 986.2 | chr2 | 38817541 | 38817960 | HNRNPLL | 196.5 |
| chr1 | 12006721 | 12007300 | PLOD1 | 728.4 | chr6 | 107020041 | 107020480 | RTN4IP1 | 826.4 |
| chr2 | 211312821 | 211313060 | LANCL1 | 2.298621746 | chr9 | 3363641 | 3364120 | RFX3 | 239.9 |
| chr8 | 28715761 | 28716500 | INTS9 | 4.201346389 | chr8 | 104412681 | 104413060 | SLC25A32 | 913.3 |
| chr7 | 74193427 | 74193460 | NCF1 | 553.5 | chr18 | 60877501 | 60877720 | BCL2 | 142.2 |
| chr1 | 1634541 | 1635120 | CDK11B | 1161.2 | chr19 | 44237601 | 44237820 | SMG9 | 174.8 |
| chr1 | 28820241 | 28820640 | PHACTR4 | 452.2 | chr6 | 143170021 | 143170620 | HIVEP2 | 6.715432961 |
| chr15 | 32823801 | 32824240 | WHAMMP1 | 10.84086799 | chr1 | 202433501 | 202433920 | PPP1R12B | 2.728413846 |
| chr12 | 120287977 | 120288079 | CIT | 3.410127991 | chr1 | 116533161 | 116533380 | SLC22A15 | 522.3 |
| chr9 | 123756161 | 123756560 | C5 | 387.7 | chr1 | 116615581 | 116615840 | SLC22A15 | 348.5 |
| chr15 | 41750021 | 41750074 | RTF1 | 163.3445378 | chr13 | 95809561 | 95810040 | ABCC4 | 3.002337442 |
| chr1 | 116620741 | 116621540 | SLC22A15 | 2.142934415 | chr17 | 59870957 | 59871090 | BRIP1 | 598.3 |
| chr13 | 95811301 | 95811660 | ABCC4 | 277.2 | chr8 | 128889621 | 128890040 | PVT1 | 1130.5 |
| chr6 | 161470161 | 161470540 | MAP3K4 | 553.5 | chr2 | 109068854 | 109068922 | GCC2 | 239.9 |
| chr8 | 38196053 | 38196125 | WHSC1L1 | 728.4 | chr3 | 57846661 | 57847000 | SLMAP | 261.7 |
| chr13 | 46577273 | 46577471 | ZC3H13 | 857.3 | chr10 | 32751944 | 32751977 | CCDC7 | 196.5 |
| chr18 | 12846781 | 12847100 | PTPN2 | 249.6 | chr10 | 93736081 | 93736460 | BTAF1 | 294.2 |
| chr6 | 155095122 | 155095206 | SCAF8 | 240.4 | chr13 | 45984381 | 45984960 | SLC25A30 | 478.9 |
| chr1 | 116552021 | 116552400 | SLC22A15 | 2.592252511 | chr11 | 88051261 | 88051720 | CTSC | 153 |
| chr3 | 155551645 | 155551680 | SLC33A1 | 148.3 | chr5 | 149401641 | 149402040 | HMGXB3 | 609.2 |
| chr5 | 79997461 | 79997820 | MSH3 | 240.4 | chr2 | 38806061 | 38806600 | HNRNPLL | 3.371440714 |
| chrX | 20211604 | 20211660 | RPS6KA3 | 332.5 | chr2 | 37291882 | 37292133 | HEATR5B | 478.9 |
| chr10 | 1104641 | 1105120 | WDR37 | 148.3 | chr1 | 1600581 | 1601020 | CDK11B | 229.1 |
| chr1 | 150197101 | 150197480 | ANP32E | 424.6 | chr6 | 143188001 | 143188480 | HIVEP2 | 174.8 |
| chr2 | 45774660 | 45774751 | SRBD1 | 719.2 | chr2 | 86074961 | 86075360 | ST3GAL5 | 294.2 |
| chr18 | 60206913 | 60207025 | ZCCHC2 | 682.4 | chr13 | 47285961 | 47285979 | LRCH1 | 153 |
| chr4 | 1913541 | 1914200 | WHSC1 | 1511.1 | chr1 | 169951681 | 169952280 | KIFAP3 | 6.078007519 |
| chr7 | 99091041 | 99091240 | ZNF394 | 4.287136929 | chr9 | 3417301 | 3417820 | RFX3 | 696.1 |
| chr6 | 64355561 | 64355940 | PHF3 | 1271.7 | chr10 | 74486801 | 74487200 | MCU | 1173.9 |
| chr6 | 107061421 | 107061820 | RTN4IP1 | 553.5 | chr5 | 80000601 | 80000980 | MSH3 | 402.8 |
| chr6 | 7304321 | 7304720 | SSR1 | 1253.3 | chr12 | 102580441 | 102580820 | PARPBP | 522.3 |
| chrX | 154020416 | 154020560 | MPP1 | 581.1 | chr9 | 37356101 | 37356500 | ZCCHC7 | 4.251028807 |
| chr9 | 123764001 | 123764380 | C5 | 175.9 | chr4 | 183828681 | 183829120 | DCTD | 674.3 |
| chr7 | 11021998 | 11022300 | PHF14 | 3785.4 | chr13 | 60485868 | 60485970 | DIAPH3 | 544 |
| chr5 | 65061921 | 65062360 | NLN | 314.1 | chr15 | 41961025 | 41961040 | MGA | 359.4 |
| chr9 | 123231821 | 123232200 | CDK5RAP2 | 470.6 | chr1 | 91403101 | 91403380 | ZNF644 | 99.11764706 |
| chr11 | 88047681 | 88048060 | CTSC | 479.8 | chr18 | 44526019 | 44526180 | KATNAL2 | 1695.2 |
| chr3 | 126738981 | 126739260 | PLXNA1 | 314.1 | chr5 | 21545161 | 21545540 | GUSBP1 | 229.1 |
| chr10 | 93736381 | 93736740 | BTAF1 | 258.8 | chr1 | 116609181 | 116609680 | SLC22A15 | 4.183759398 |
| chr8 | 48955641 | 48955741 | UBE2V2 | 2.998688008 | chr9 | 123799601 | 123800980 | C5 | 1955.9 |
| chr1 | 202434741 | 202435140 | PPP1R12B | 452.2 | chr2 | 203817501 | 203817960 | WDR12 | 1467.2 |
| chr8 | 120842381 | 120843080 | TAF2 | 1391.4 | chr6 | 117019866 | 117019961 | KPNA5 | 565.7 |
| chr12 | 102530201 | 102530440 | PARPBP | 415.3 | chr22 | 32168721 | 32169102 | DEPDC5 | 565.7 |
| chr17 | 28037561 | 28037860 | SSH2 | 203.6 | chr2 | 32620981 | 32621300 | BIRC6 | 533.2 |
| chr2 | 100081383 | 100081447 | REV1 | 1244 | chr1 | 1626641 | 1627060 | CDK11B | 522.3 |
| chr2 | 203825321 | 203825720 | WDR12 | 222 | chr18 | 12844381 | 12844760 | PTPN2 | 261.7 |
|  |  |  |  |  | chr1 | 45167301 | 45167520 | G002087 | 370.3 |
|  |  |  |  |  | chr3 | 49419341 | 49419620 | RHOA | 739.5 |
|  |  |  |  |  | chr3 | 57833881 | 57834580 | SLMAP | 793.8 |
|  |  |  |  |  | chr2 | 206930081 | 206930720 | INO80D | 1423.7 |
|  |  |  |  |  | chr16 | 21828782 | 21828839 | RRN3P1 | 2933.3 |
|  |  |  |  |  | chr6 | 107046461 | 107046840 | RTN4IP1 | 457.1 |
